# Sugammadex and Ideal Body Weight in Bariatric Surgery

**DOI:** 10.1155/2013/389782

**Published:** 2013-06-06

**Authors:** Maria Sanfilippo, Francesco Alessandri, Ahmed Abdelgawwad Wefki Abdelgawwad Shousha, Antonio Sabba, Alessandra Cutolo

**Affiliations:** Department of Anesthesiology and Intensive Care, Sapienza University, Viale del Policlinico 155, 00161 Rome, Italy

## Abstract

*Background*. The obese patients have differences in body composition, drug distribution, and metabolism. Sugammadex at *T*_2_ recovery in a dose of 2 mg kg^−1^ of real body weight (RBW) can completely reverse the NMB block; in our study we investigated the safety and efficacy of Sugammadex dose based on their ideal body weight (IBW). *Methods*. 40 patients of both sexes undergoing laparoscopic bariatric surgery were enrolled divided into 2 groups according to the dose of Sugammadex: the first received a dose of 2 mg kg^−1^ of IBW and the second received a dose of 2 mg kg^−1^ of RBW. Both were anesthetized with doses calculated according to the IBW: fentanyl 2 **μ**g kg^−1^, propofol 3 mg kg^−1^, rocuronium 0,6 mg kg^−1^, oxygen, air, and desflurane (6–8%). Maintenance doses of rocuronium were 1/4 of the intubation dose. Sugammadex was administrated at *T*_2_ recovery. *Results*. The durations of intubation and maintenance doses of rocuronium were similar in both groups. In IBW group, the *T*_4_/*T*_1_ value of 0.9 was reached in 151 ± 44 seconds and in 121 ± 55 seconds in RBW group (*P* = 0.07). *Discussion*. Recovery times to *T*_4_/*T*_1_ of 0.9 are surprisingly similar in both groups without observing any postoperative residual curarization. *Conclusion*. Sugammadex doses calculated according to the IBW are certainly safe for a rapid recovery and absence of PORC.

## 1. Introduction

Sugammadex, a modified *γ*-cyclodextrin, has been recently introduced into clinical practice as a selective relaxant binding agent for antagonism of prolonged rocuronium-induced neuromuscular block during general anaesthesia.

Sugammadex forms a complex with the neuromuscular blocking agents (NMBAs) rocuronium or vecuronium. By forming complexes with these NMBAs, it reduces their ability to bind to nicotinic receptors at the neuromuscular junction. Upon injection of Sugammadex, any rocuronium or vecuronium molecules present in the plasma and at their sites of action are attracted to Sugammadex via lipophilic binds in a ratio of one Sugammadex molecule to one molecule of the NMBA [1–4]. This causes a concentration gradient and any remaining rocuronium or vecuronium molecules are attracted back into the plasma and become bound to free Sugammadex molecules [2, 3].

 At *T*_2_ recovery of train-of-four (TOF) Sugammadex can completely reverse the neuromuscular block in a dose of 2 mg kg^−1^ of real body weight (RBW). Obesity does not affect the efficacy of Sugammadex in reversing rocuronium-induced neuromuscular block [5], but the majority of authors and the literature recommend Sugammadex dose to be calculated upon RBW. 

As the obese patients have a different drug distribution and body composition, we investigated the safety and efficacy of Sugammadex according to the ideal body weight (IBW). 

## 2. Materials and Methods

After the Ethic Committee approval and informed consent, forty patients of both sexes, aged between 18–54 years, ASA II-III, with a BMI > 38 kg/m^2^ scheduled for elective laparoscopic bariatric surgery (sleeve gastrectomy) under general anaesthesia, were enrolled in this study. Exclusion criteria were chronic alcoholism or drug abuse, liver and renal dysfunction, disabling neuropsychiatric disorders, history of stroke, brain trauma in the last 12 months, hypersensitivity to anesthetics, history of myocardial infarction, adrenal insufficiency, congestive heart failure, and lack of cooperation or legal incapacity. Patients with obstructive sleep apnea syndrome were also excluded. All patients were subdivided into two groups according to the administrated dose of Sugammadex: IBW group had received a dose of 2 mg kg^−1^ of ideal body weight (IBW), calculated from Broca's formula (Height in cm-106), and RBW group received a dose of 2 mg kg^−1^ of RBW. They were all anesthetized according to their IBW with fentanyl 2 *μ*g kg^−1^, propofol 3 mg kg^−1^, rocuronium 0,6 mg kg^−1^, oxygen, air, and desflurane (6–8%). Maintenance doses of rocuronium were calculated as 1/4 of the intubation dose and were administrated at *T*_2_ recovery. At the end of surgery, Sugammadex was administrated at *T*_2_ recovery. All patients were monitored for electrocardiogram, invasive blood pressure, heart rate, end tidal CO_2_ concentration, oxygen saturation, and diuresis. The depth of anaesthesia was monitored with BIS (BIS VISTA QUATRO SISTEM). Neuromuscular function was evaluated with TOF GUARD; body temperature was maintained at 36°-37°C.

Hemogasanalysis was performed preoperatively, 20 min after extubation with a FiO_2_ of 40%, and 120 min after extubation in air.

Invasive blood pressure measurement was established by placing a cannula needle in the radial artery.

After induction of anaesthesia, neuromuscular monitoring was carried out using the acceleromyography of the adductor pollicis muscle. Single twitch stimulation of the ulnar nerve has been performed until we obtained the 100% of muscle response; then the monitoring was switched to TOF mode (70 mA current, 0.2 ms pulse duration, 2 Hz frequency) every 12 sec. After 2 minutes of stabilization of the acceleromyography records, 0.6 mg kg^−1^ of rocuronium was injected in 10 seconds. The endotracheal intubation was carried out when *T*_1_ was absent [6]. 

During surgery, the maintenance doses of rocuronium were injected at *T*_2_ recovery (in the TOF corresponding about the 25% of muscle recovery). Neuromuscular function monitoring was continued until the end of surgical procedure and at least 10 min after the TOF ratio of 0.9.

 Anaesthesia was maintained by desflurane together with opioids. At the end of surgery and emergence of anaesthesia at *T*_2_ recovery, IBW group patients received a bolus dose of Sugammadex according to their IBW, while the RBW group received a bolus dose of Sugammadex according to their RBW. 

All patients were extubated at TOF ratio of 0.9. They were monitored in the recovery room for 120 min after extubation. Oxygen saturation, respiratory rate, heart rate, and blood pressure were routinely monitored. 

For postoperative pain control we used in both groups a nonsteroidal anti-inflammatory drugs (Ketorolac 60 mg IV) and Paracetamol 500 mg/IV (repeated every 6 hours). 

The patients were also monitored for the appearance of any sign of reoccurrence of muscle weakness such as difficult swallowing, diplopia, blurred vision, and respiratory difficulties. The neurological clinical tests (for being awake, oriented for time and place, being aroused with minimal stimulation, ability to swallow, and ability for a clear vision) were performed every 20 min and before discharge from the postoperative recovery room. 

All the 40 patients were transferred to their regular surgery wards.

The primary endpoint in this study was to evaluate the recovery times to *T*_4_/*T*_1_ of 0.9 after the administration of the Sugammadex based on the IBW in bariatric surgery. 

Data were analysed as mean values (MV) ± standard deviation (SD). Two-tailed Student's *t*-test for paired data was used for calculation of statistical significance (*P* < 0.05).

## 3. Results

The total 40 patients enrolled in this study were comparable with respect to age, height, weight, BMI, and ASA. (Table 1).

The BIS values were recorded in preinduction phase, during intubation, 5 minutes after the administration of Sugammadex, and, lastly, at extubation (Table 2).

From Table 2 we concluded that there are no significant statistical difference between the two groups regarding the BIS levels after the administration of the Sugammadex different doses (*P* value > 0.05).

**Figure 1:**
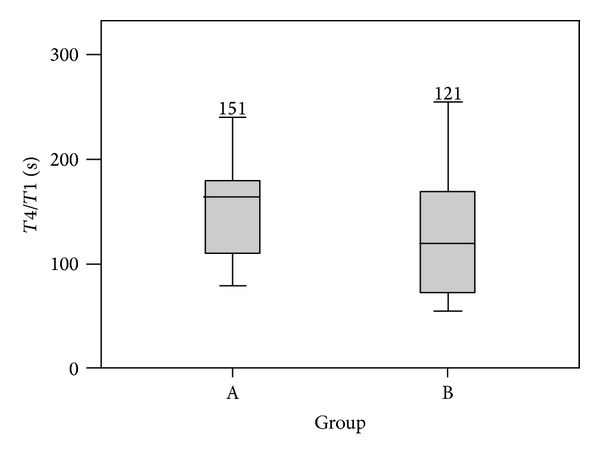
*T*_4_/*T*_1_ recovery (sec) after bolus dose of Sugammadex in groups IBW (A) and RBW (B).

The median time for recovery to TOF ratio of 0.9 was recorded at 30 seconds intervals after the injection of Sugammadex. It was about 151 ± 44 sec in IBW group and of 121 ± 55 sec in RBW group (*P* = 0.07) (Figure 1-Table 3).

The hemogasanalysis values are shown in Table 4. 

There was no significant difference of hemogasanalysis values between the two groups; however, the slight increase of pCO_2_ observed 20 min after extubation was correlated to the abdominal insufflation of CO_2_ throughout the surgical procedure, and simply it required time to be eliminated. In fact, at 120 min after extubation, the pCO_2_ values returned to the preoperative levels.

No signs of residual neuromuscular block were observed in any patient during the clinical assessment in the recovery room. Clinical tests and evaluation of consciousness did not reveal any difference in both groups. 

None of the patients presented respiratory events or had a need for a respiratory support following extubation. The most frequently observed events were postoperative nausea and vomiting (PONV) (18 patients) treated with 4 mg Ondansetron or 2.5 mg Droperidol, postoperative shivering (10 patients) treated with 30–60 mcg clonidine. 

## 4. Discussion

The incidence of morbid obesity (BMI > 40 kg·m^−2^) in western populations is 2–5%. The introduction of bariatric surgery has been a considerable breakthrough in the treatment of these patients [7].

 For such patients, calculation of an appropriate drug dose is a problem. Pharmacokinetic studies show that weakly lipophilic drugs such as rocuronium should be dosed on ideal body weight (IBW), rather than real body weight (RBW) [8]. The pharmacokinetic profile of Sugammadex is similar to that of rocuronium, despite the fact that Sugammadex has no affinity for plasma proteins.

 Sugammadex dosage is usually based on RBW without taking fat content into consideration. 

This study was definite to assess the efficacy and safety of Sugammadex in a dose of 2 mg kg^−1^ to obtain the reversal of rocuronium-induced neuromuscular block specifically in severely obese patients. Obesity is associated with changes in body composition and function that may alter drug distribution and metabolism. Cardiac output (COP), glomerular filtration rate (GFR), and intravascular volume are all increased [9, 10]. In addition, there is evidence that obesity alters both liver function and protein binding [11, 12].

The main risk factors affecting the process of tissue diffusion of drugs are body composition, plasma, protein binding, and regional blood flow [13], and in the case of muscle relaxants, these parameters correlate to the biophase in which these drugs act. It should be noted that a drug's measured molar potency is the end result of many contributing factors: the drug's intrinsic potency (the CE50, or the biophase concentration resulting in 50% twitch depression), the rate of equilibration between plasma and the biophase (ke0), the initial rate of plasma clearance, and probably other factors as well [14, 15]. Furthermore the postulated existence of a volume called “apparent space of interaction” seems to be the final target of muscle relaxants [16]. The Lean Body Mass (LBM) represents muscle relaxants biophase in its totality, that is, the junctional pre- and postsynaptic membrane, the intersynaptic fluid, interstitial space volume, and its vessels. The right calculation of the LBM allows us to administer the right dose of muscle relaxant. In the case of muscle relaxant, the differences in results are probably due to the method of dose calculation because it is considered the total body weight (TBW) or the ideal body weight (IBW). The reduced total dose of rocuronium that we used in both groups has produced 100% of neuromuscular block and good muscle relaxation throughout the surgical procedure, once more confirming that in obese patients the muscle relaxant should be administered according to the ideal body weight [17].

Since 2008, Sugammadex, a specific encapsulator of steroid muscle relaxants rocuronium and vecuronium, is available in our country. Previous dose-finding studies were restricted to reversal of profound deep or moderate rocuronium neuromuscular block [18–21]. For moderate neuromuscular block, defined by the presence of two TOF responses, the Sugammadex dose of 2 mg kg^−1^ is recommended [22]. 

The introduction of new drugs with new mechanisms of action should be carefully monitored before arriving to enthusiastic results. 

The under-dosing of Sugammadex may lead to reappearance of neuromuscular block after apparent successful recovery. Eleveld et al. were the first to describe this phenomenon and they supposed that recurarization occurred because of too small dose of Sugammadex for the given degree of block [23].

In small doses, Sugammadex may form complexes only with molecules of rocuronium in the central compartment and cannot sustain the redistribution from peripheral to central compartments. Drobnik et al. administered both suboptimal and an adequate dose of Sugammadex; they observed an incomplete reversal and recurarization in patients receiving suboptimal dose and full recovery in those receiving the recommended dose of 4 mg kg^−1^ [24].

Our Study showed that during desflurane anaesthesia with a TOF ratio higher than 0.9 occurred within 1.5 min, Sugammadex can provide a rapid and safe reversal of moderate neuromuscular block after administration of rocuronium dose of 0.6 mg kg^−1^ (based on IBW) in morbidly obese patients.

Sugammadex was well tolerated by all patients and we did not observe any adverse events and our profile was consistent with previous studies [25]. 

In conclusion, Sugammadex in a dose of 2 mg kg^−1^ of IBW facilitated rapid recovery from moderate rocuronium-induced neuromuscular block in morbidly obese patients.

## Figures and Tables

**Table 1:** Population.

	IBW group (*n* = 20)	RBW group (*n* = 20)
Age (yrs)	32.2 ± 6.1	30.6 ± 7.4
Real body weight (Kg)	121.5 ± 12.3	125.3 ± 14.7
Ideal body weight (Kg)	65.3 ± 8.5	64.5 ± 7.3
Height (cm)	165.3 ± 8.5	164.5 ± 7.4
Body mass index (kg/m^2^)	44.5 ± 4.2	46.47 ± 4.7
ASA (II/III)	18/2	15/5
Duration of surgery (min)	108.1 ± 27.3	103.7 ± 32.1

**Table 2:** BIS level in both groups.

	IBW group	RBW group
Preinduction	97.5 ± 0.452	97.7 ± 0.178
Intubation	31 ± 3.65	32 ± 2.47
5 min after Sugammadex dose	62.4 ± 1.254	63.1 ± 2.024
Extubation	84.9 ± 2.78	85.4 ± 1.62

**Table 3:** Time for *T*_4_/*T*_1_ recovery in IBW and RBW groups.

Time in seconds	IBW	RBW
30	0.34 ± 0.11	0.42 ± 0.13
60	0.45 ± 0.23	0.55 ± 0.10
90	0.71 ± 0.08	0.72 ± 0.02
120	0.88 ± 0.1	0.90 ± 0.07
150	0.90 ± 0.57	0.91 ± 0.08

**Table 4:** Hemogasanalysis in IBW and RBW groups.

	Preoperative	20 min after extubation	120 min after extubation
IBW group			
pH	7.42 ± 0.03	7.31 ± 0.02	7.42 ± 0.03
pO_2_	93.5 ± 3.4	107.2 ± 3.2	95.4 ± 3.1
pCO_2_	35.8 ± 12.5	42.2 ± 21.4	36.0 ± 15.1
HCO_3_^−^	23.3 ± 1.7	21.3 ± 2.2	23.1 ± 1.4
SpO_2_	98.4 ± 0.71	98.5 ± 2.3	98.1 ± 1.5
RBW group			
pH	7.42 ± 0.02	7.3 ± 0.04	7.4 ± 0.03
pO_2_	90.5 ± 2.48	135.5 ± 5.5	94.0 ± 2.7
pCO_2_	37.8 ± 10.7	44.6 ± 35.4	37.7 ± 8.9
HCO_3_^−^	24.4 ± 1.7	22.6 ± 1.8	24.1 ± 2.2
SpO_2_	97.8 ± 1.6	99.3 ± 0.5	98.0 ± 1.0
